# Lymphocyte signal transduction defects in cutaneous sarcoidosis and implications for treatment

**DOI:** 10.3389/fimmu.2026.1711377

**Published:** 2026-09-08

**Authors:** Julia Lunt, Adarsh Shidhaye, Christine Fasana, Meredith Morgan, William M. Schmidt, Daniel L. Lee, Sherry Chen, Katherine E. McCain, Christine Schammel, Steven E. Fiester, Sergio Arce, Jennifer T. Grier

**Affiliations:** 1 Department of Biomedical Sciences, University of South Carolina School of Medicine Greenville, Greenville, SC, United States; 2 Department of Biological Sciences, University of South Carolina, Columbia, SC, United States; 3 Department of Pathology, Prisma Health, Greenville, SC, United States; 4 Pathology Associates and Consultants, Greenville, SC, United States; 5 Department of Biological Sciences, Florida Gulf Coast University, Fort Myers, FL, United States; 6 Department of Chemistry, Furman University, Greenville, SC, United States; 7 School of Health Research, Clemson University, Clemson, SC, United States; 8 Family Medicine Residency at Lee Health, Florida State University College of Medicine, Fort Myers, FL, United States; 9 Internal Medicine Residency at Lee Health, Florida State University College of Medicine, Cape Coral, FL, United States; 10 Academic Medicine Enterprise, Lee Health, Fort Myers, FL, United States; 11 Prisma Health Cancer Institute, Prisma Health System, Greenville, SC, United States

**Keywords:** cutaneous sarcoidosis, cutaneous sarcoidosis treatment, granulomas, granulomatous disease, non-caseating granulomas, sarcoidosis

## Abstract

Sarcoidosis is a multiorgan immunological disease characterized by an exaggerated inflammatory response leading to non-caseating granuloma formation. Cutaneous sarcoidosis lesions serve as an accessible site for biopsy that can assist with earlier identification and treatment of sarcoid disease. This review synthesizes recent advances in the understanding of cutaneous sarcoidosis immunopathogenesis, with a focus on lymphocyte signaling, and describes the therapeutic implications of this immune dysregulation. While it is known that Th1 and Th17 cell subsets contribute to general non-caseating granuloma formation, recent studies identify Th17.1 cells as the predominant producers of IFN-γ, challenging classical paradigms. Th2 polarization and elevated thymus and activation-regulated chemokine (TARC) levels, along with regulatory T cell dysfunction and PD-1 overexpression, contribute to chronic inflammation and fibrosis in the cutaneous sarcoidosis disease state. The JAK/STAT pathway is notably upregulated in cutaneous sarcoid granulomas, and JAK inhibitors have demonstrated promising efficacy as an avenue for treatment. Additional immune mechanisms at play in cutaneous sarcoidosis include mTOR and TLR dysregulation as well as B-cell dysfunction. Infectious triggers and DNA polymorphisms associated with genetic susceptibility further support the multifactorial nature of cutaneous sarcoidosis. Based on current literature and clinical guidelines, we propose treatment options for cutaneous sarcoidosis which target dysregulated aspects of the immune response. While prednisone and corticotropin gel remain the only FDA-approved therapies for cutaneous sarcoidosis, improved understanding of sarcoidosis signaling may lead to promising new therapeutic approaches. With the growing understanding of lymphocyte signaling defects in cutaneous sarcoidosis, physicians will be better equipped to identify and treat the underlying mechanisms of this disease.

## 1 Introduction

Sarcoidosis is characterized by an exaggerated inflammatory response that consists of dysregulated innate and adaptive immune responses resulting in multiorgan immune-mediated non-caseating granuloma formation. The prevalence is estimated at 1–35 per 100,000 individuals with an associated mortality rate of 1-5% (1). The lungs, lymphatic system, eyes and skin are most commonly affected, with cutaneous involvement occurring in 20-30% of patients (2). Clinically, patients may present with pulmonary symptoms such as dry cough, wheezing, dyspnea, and chest tightness, as well as cutaneous lesions, renal symptoms (renal sarcoidosis), and/or neurological involvement (neurosarcoidosis); however some patients may remain asymptomatic (1).

**Figure 1:**
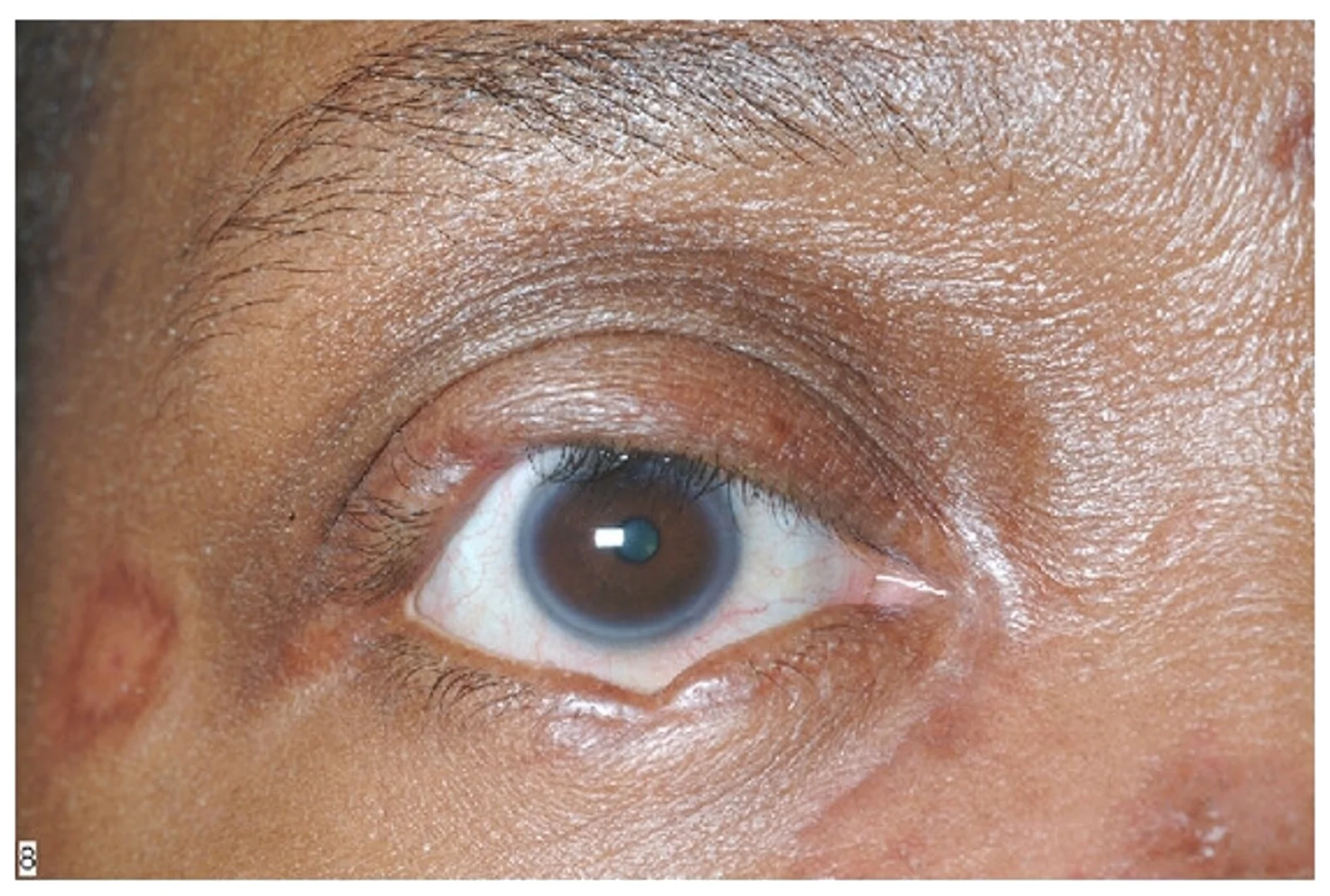
Clinical image of eyelid notching and scarring following a 5-year history of eyelid nodules, with additional sarcoid nodules present on the face. The patient was previously diagnosed with severe systemic sarcoidosis including cutaneous, ocular, and pulmonary involvement. Reproduced from Collins ME, Petronic-Rosic V, Sweiss NJ, Marcet MM. Full-thickness eyelid lesions in sarcoidosis. Case Rep Ophthalmol Med. (2013) doi: 10.1155/2013/579121 under a Creative Commons CC-BY 3.0 license.

Cutaneous sarcoidosis most frequently presents with papules and plaques on the face, but may occur at any site and display a wide range of morphologies (3). Papules and nodules of cutaneous sarcoidosis characteristically involve the eyelids and nasolabial folds (**Figure 1**), and appear as firm, flesh-colored, red-brown or violaceous raised lesions, that are either localized or generalized (3, 4). They often exhibit a translucent yellow-brown hue with surrounding erythema and may be umbilicated or assume an annular shape (5). Cutaneous sarcoidosis can also present as macules, subcutaneous nodules, scar sarcoidosis, or lupus pernio, and may be associated with scars, tattoos, and injuries (5, 6). Less commonly, presentations of cutaneous sarcoidosis include ichthyosiform sarcoidosis, atrophic and ulcerative sarcoidosis, nail sarcoidosis, mucosal sarcoidosis, and lymphedematous sarcoidosis (5). Cutaneous sarcoidosis may represent the initial manifestation of systemic sarcoidosis and is frequently indicative of systemic disease burden, thus cutaneous sarcoid lesions may provide clinically relevant information regarding overall disease activity (3).

Recognition of potential cutaneous sarcoidosis is crucial as the skin lesions are readily accessible for biopsy, facilitating disease diagnosis and initiation of therapy. Identification of cutaneous granulomatous lesions can be complemented by other clinical features of systemic inflammation to aid in distinguishing cutaneous or systemic sarcoid disease from sarcoid mimics or isolated cutaneous granulomas. Clinical testing to supplement skin biopsy in the diagnosis of sarcoidosis would include chest X-ray, computed tomography, and pulmonary function tests, as well as serum proteins and Calcium (Ca^2+^), with the presence of mediastinal and/or hilar lymphadenopathy, decreased lung function, polyclonal gammopathy, and elevated serum ACE and/and Ca^2+^ suggestive of sarcoidosis (7–9). Immune investigation of cutaneous lesions may also offer a clinically useful window into underlying systemic inflammation. Experimental models using intradermal injection of Kveim-Siltzbach antigen, sarcoid spleen extract, demonstrated that non-caseating granuloma formation in sarcoidosis reflects a hyperactive, dysregulated immune response to antigen stimulation. In this model, patients developed a papule at the injection site 3–6 weeks after injection, which was histologically characterized as non-caseating granulomatous inflammation. Pathophysiological activation of T lymphocytes, cytokines, and regulatory cells, as well as additional immunologic pathways were found to have led to granulomatous inflammation and the associated cutaneous changes (10). This enhanced understanding of non-caseating granuloma pathogenesis in sarcoidosis demonstrated that cutaneous sarcoid manifestations may serve as a reflection of an underlying immune system dysfunction in the skin and/or body (6).

Cutaneous sarcoid lesions may also provide a non-invasive method for disease monitoring after diagnosis as specific morphologic subtypes of cutaneous lesions serve as clinically meaningful indicators of the severity and chronicity of internal organ involvement (**Table 1**) (3). For example, papules and maculopapular eruptions typically represent more superficial, less fibrotic granulomatous inflammation and are often seen in acute or early disease (11). These lesions are more commonly associated with limited systemic involvement and carry a favorable prognosis, frequently responding to topical or intralesional corticosteroids and antimalarials, with a higher likelihood of spontaneous or treatment-induced remission (12–14). On the other hand, lesions such as lupus pernio and infiltrated plaques are associated with an increased risk of advanced or persistent systemic disease, whereas scar and tattoo sarcoidosis reflect a localized, antigen-driven granulomatous response at sites of prior trauma or pigment deposition (15–17). Although sometimes limited, scar and tattoo sarcoidosis may be the first manifestation of systemic disease and should prompt evaluation for internal organ involvement (18, 19).

**Table 1:** Cutaneous sarcoidosis clinical and immunological characteristics.

Lesion type	Clinical features	Immunologic highlights	Systemic correlation	Treatment response
Papules	small, firm, red-brown, often symmetric	superficial granulomas; mild CD4^+^ T-cell infiltrate (11)	usually mild/limited systemic disease (12, 13)	good response to topical/intralesional therapy; may remit spontaneously (13, 14)
Plaques	larger, indurated, chronic	dense dermal granulomas; fibrosis (5)	associated with chronic systemic involvement (3, 15)	often requires systemic therapy; slower, less complete response (3, 4, 13)
Lupus Pernio	chronic, violaceous, disfiguring; face, nose, lips	dense granulomas with fibrosis; persistent Th1/Th17 activity (37)	strong marker of severe systemic disease (3, 15, 16)	poor response to local therapy; needs aggressive systemic treatment (3, 16)
Scar/Tattoo	raised, firm, violaceous at scar/pigment sites	granulomas triggered by local antigen; similar Th1/Th17 profile (17)	variable systemic association; may precede systemic disease (18, 19)	often responds to intralesional steroids; systemic therapy if systemic disease active (17, 18)
Maculopapular	small erythematous/red-brown macules	superficial granulomas; mild inflammation (11)	typically acute, limited disease (3, 11)	good response to topical therapy; may resolve spontaneously (3, 15)

**Figure 2:**
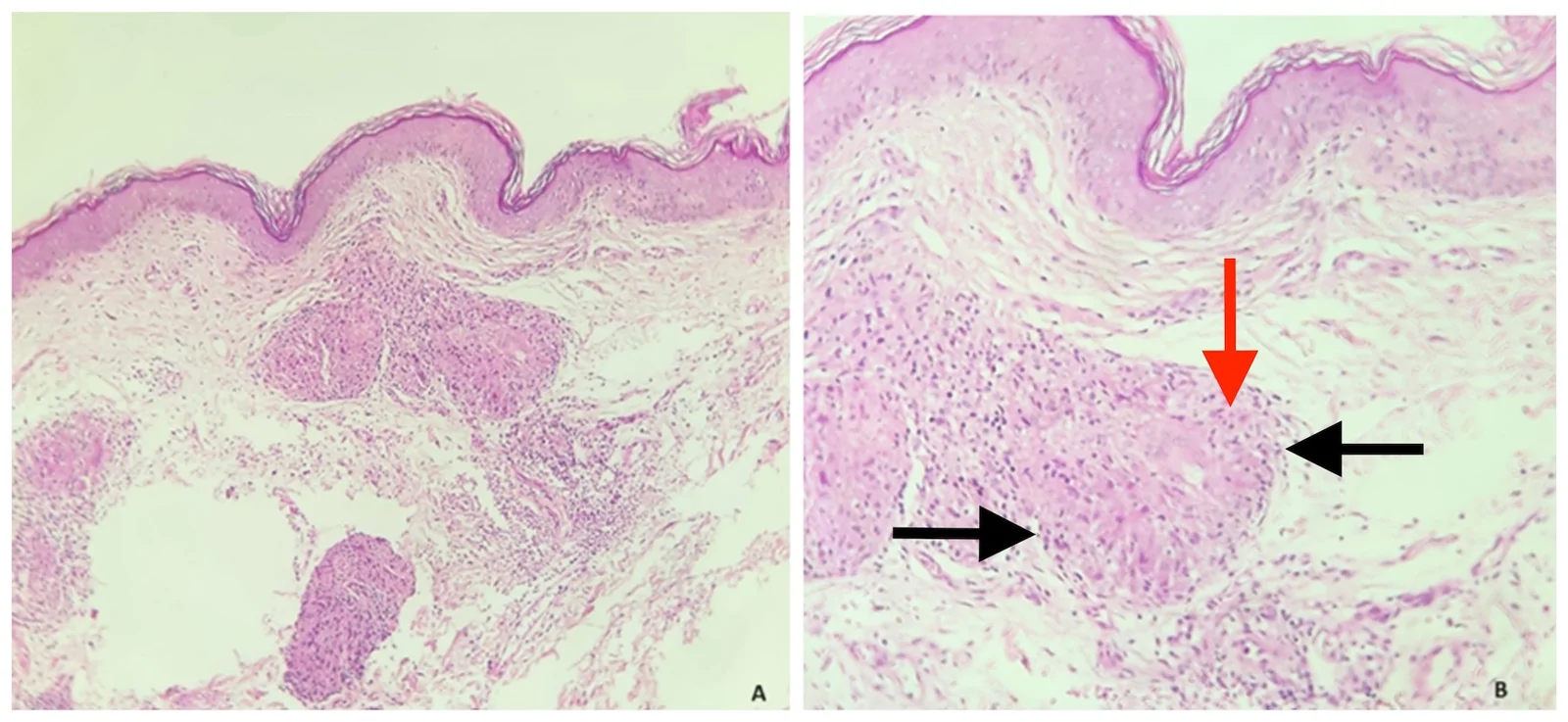
Well-formed non-caseating granulomas of epithelioid histiocytes and giant cells associated with sparse lymphocytic inflammation without necrosis. H&E stain. **(A)** 200X, **(B)** 400X. Lymphocyte infiltration is indicated by black arrows, while the red arrow identifies a multinucleated giant cell adapted from Tlemcani, et al., 2024 (158) under a Creative Commons CC-BY 4.0 license.

Efforts are being made to better understand the clinical distinctions between cutaneous manifestations in systemic sarcoidosis and purely cutaneous sarcoidosis. Cutaneous sarcoidosis demonstrates the same fundamental histopathologic feature of non-caseating granuloma formation as sarcoid lesions in other organs (e.g., lung, spleen) (**Figure 2**) (20). Similarities between systemic and cutaneous sarcoidosis extends to both innate and adaptive cellular participants, as well as to the principal cytokines, mediators, and molecular pathways involved. Both systemic and cutaneous disease states follow a similar mechanism when forming non-caseating epithelioid granulomas, mediated by Th1 cells, and the lesions are considered immunologically equivalent (21, 22). Common histopathologic features of sarcoid granulomas include an aggregation of multinucleated giant cells and epithelioid cells, of monocytic origin, within the follicular region of the granuloma and a region of lymphocytic cell accumulation in the surrounding tissue (**Figure 2B**) (23). Despite similarities in the mediators and components underlying cutaneous and systemic sarcoidosis, subtle distinctions exist. These differences relate primarily to the intensity and distribution of the lymphocytic infiltrate with a higher proportion of CD4^+^ T cells in pulmonary granulomas relative to cutaneous granulomas, variation in circulating biomarkers such as greater elevations in C-reactive protein in systemic versus cutaneous disease, and differential treatment responses with more favorable response rates for isolated cutaneous sarcoidosis than systemic forms (24). One potential source of these immunological variations may be the site of antigen exposure. While the lungs are thought to be the main site of antigen exposure in pulmonary sarcoidosis, the high prevalence of skin manifestations in non-pulmonary sarcoidosis suggests that the skin may be a predominant site of antigen exposure leading to cutaneous-specific granuloma formation (25).

Herein we review the current understanding of the immunologic pathogenesis of cutaneous sarcoidosis and delineate how dysregulated lymphocyte signaling contributes to granuloma formation and disease progression (**Table 2**). This narrative review is primarily focused on cutaneous sarcoidosis, however evidence from systemic or pulmonary sarcoidosis is described in cases where cutaneous-specific information is incomplete or absent. We further examine the clinical implications of this immune dysregulation and evaluate the potential role of immunomodulatory therapies in the management of cutaneous sarcoidosis. Review of the literature was completed utilizing PubMed and the search terms included “sarcoidosis”, “cutaneous sarcoidosis”, “sarcoidosis treatments”, and “cutaneous sarcoidosis treatments”. Selected citations include studies available in English and articles from the past 50 years, with a preference for articles published in the last 25 years. This detailed characterization of the altered lymphocyte signaling pathways in cutaneous sarcoidosis can serve as a guide for elucidating disease evolution and identifying novel therapeutic approaches.

**Table 2:** Signaling dysregulation in cutaneous sarcoidosis.

Cell type/pathway	Key mediators/markers	Evidence of involvement in cutaneous sarcoidosis	Evidence in systemic or pulmonary sarcoidosis	Potential targeted therapeutics
Th1/Th17/Th17.1 cells	IFN-γ, IL-17A, IL-6, STAT1, STAT3	IL-17A, *IL-6*, and *IFN-γ* overexpression and STAT1/STAT3 activation in skin lesions; reduction of *IL-6* and *IFN-γ*, expression correlates with clinical improvement (34, 35, 43)	expanded Th17.1 cells produce IFN-γ; IL-17A^+^IFN-γ^+^ memory T cells elevated in lesions and BAL; *IL-6, IFN-γ*, and STAT1/STAT3 overexpression (32, 33, 36, 38)	JAK/STAT inhibitors (tofacitinib, ruxolitinib, baricitinib);
Th2 cells	IL-4, IL-13, TARC		TARC expressed in skin biopsies and elevated in serum of systemic sarcoidosis; Elevated Th2:Th1 ratio linked to severity (50)	investigational Anti–IL-4/IL-13 agent (dupilumab) (153)
Regulatory T cells (Tregs)	FOXP3, PD-1	increased Tregs in granulomas (54), FOXP3 enhancer-associated polymorphism protective against cutaneous granulomas (62)	reduced Treg suppressive function associated with disease; PD-1^+^ CD4^+^ T cells linked to impaired proliferation and fibrosis via IL-17A/TGF-β (56–58, 61, 63, 65, 66)	PD-1 inhibitor (pembrolizumab)
mTOR/autophagy dysregulation	mTORC1, mTORC2, Rac1, RhoA, TSC2,	inhibition in human trial decreases or resolves lesions (85)	activation promotes non-caseating granuloma formation; Inhibition reverses lesions in murine models (72, 80, 84)	mTOR inhibitors (sirolimus, everolimus, rapamycin)
TLR signaling	TLR2, TLR3, TLR4, TLR5, TLR6, TLR7, TLR8; NF-κB (p65)	overexpression of multiple TLRs in lesions, especially TLR5 and TLR6; NF-κB activation in granulomas (91)		TLR antagonists (TLR2, TLR4, TLR7, TLR9 inhibitors); NF-κB pathway inhibitors
B-cell/humoral immunity	CD19^+^, CD27^+^ memory B cells, BAFF	BAFF elevated in granulomas (94)	dysregulated antibody production; B-cell lymphopenia and reduced CD19^+^ and CD27^+^ memory B cells; BAFF elevated in serum correlating with severity (93–96)	anti-CD20 (rituximab), anti-BAFF (belimumab)
Infectious agents	mycobacterium spp. and cellular products	detected in skin lesions; improvement with antimycobacterial therapy (107–110)		antimycobacterial regimens
DNA polymorphisms	*IL23R, CCDC88B, TYK2, ANXA11, NOD2, HLA-DRB1, HLA-DQB1*		multiple GWAS/TWAS loci linked to sarcoidosis; *IL-23R locus*, *CDC88B* and *TYK2* gene expression linked to disease; specific HLA alleles linked to cutaneous disease (113–115)	future potential for genotype-guided therapy

ANXA11, annexin A11; BAFF, B-cell activating factor; BAL, bronchoalveolar lavage; CCDC88B, coiled-coil domain containing protein 88B; CD4+, CD4-positive T lymphocytes; CD19, cluster of differentiation 19; CD20, cluster of differentiation 20; CD27, cluster of differentiation 27; FOXP3, forkhead box P3; GWAS, genome-wide association study; HLA-DRB1, human leukocyte antigen DR beta 1; HLA-DQB1, human leukocyte antigen DQ beta 1; IFN-γ, interferon gamma; IL-4, interleukin-4; IL-6, interleukin-6; IL-13, interleukin-13; IL-17A, interleukin-17A; IL-23R, interleukin-23 receptor; JAK, Janus kinase; mTOR, mechanistic target of rapamycin; mTORC1, mTOR complex 1; mTORC2, mTOR complex 2; NF-κB, nuclear factor kappa-light-chain-enhancer of activated B cells; NOD2, nucleotide-binding oligomerization domain-containing protein 2; p65, RelA subunit of NF-κB; PD-1, programmed cell death protein 1; Rac1, Ras-related C3 botulinum toxin substrate 1; RCT, randomized controlled trial; RhoA, Ras homolog family member A; STAT1, signal transducer and activator of transcription 1; STAT3, signal transducer and activator of transcription 3; TARC, thymus and activation-regulated chemokine (CCL17); TGF-β, transforming growth factor beta; Th1/2/17, T helper cell subsets; Th17.1, interferon-γ–producing Th17 subset; TLR, Toll-like receptor; TLR2/3/4/5/6/7/8/9, individual Toll-like receptor family members; Tregs, regulatory T cells; TSC2, tuberous sclerosis complex subunit 2; TWAS, transcriptome-wide association study; TYK2, tyrosine kinase 2.

## 2 Immunopathology in cutaneous sarcoidosis and contemporary therapy

**Figure 3:**
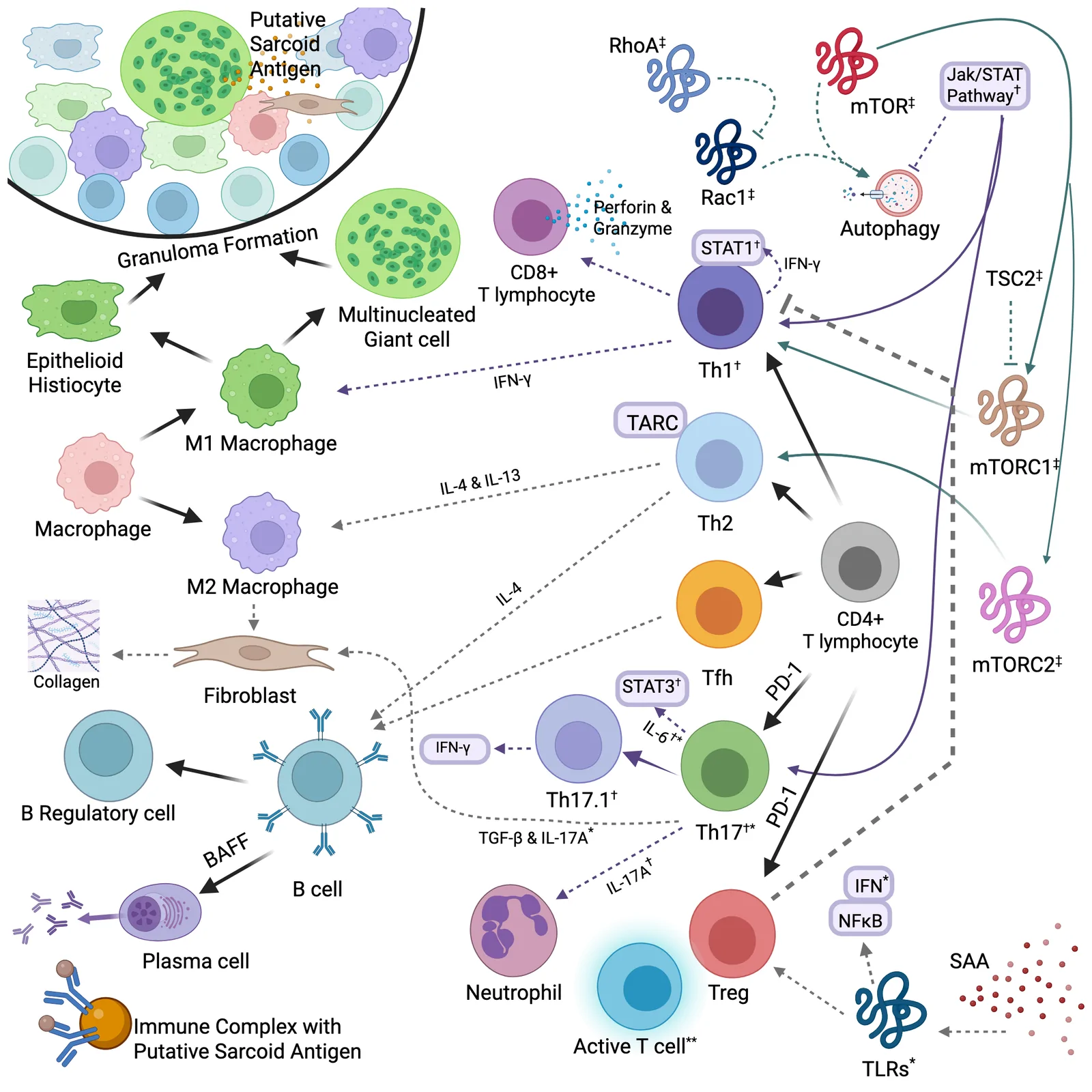
Cellular signaling involved in cutaneous sarcoidosis. Persistent antigens engage TLR2–8 on keratinocytes and dermal immune cells, activating NF-κB and type I IFN pathways and inducing IL-6 and IFN-γ. These signals drive JAK/STAT activation (STAT1/STAT3 phosphorylation) in naïve CD4^+^ T cells, promoting Th1, Th17, and Th17.1 polarization with production of IFN-γ and IL-17A. Crosstalk between these T-cell subsets and macrophages leads to non-caseating granuloma formation, composed of epithelioid macrophages and multinucleated giant cells surrounded by CD4^+^ T cells; IFN-γ–mediated macrophage activation and M1–M2 polarization influence inflammatory versus fibrotic outcomes. Chronic disease features a Th2 shift, increased TARC (CCL17), Treg dysfunction, PD-1 upregulation with IL-17/TGF-β–driven fibrosis, and B-cell abnormalities (memory B-cell depletion, elevated BAFF). Together, these pathways govern granuloma formation, lesion phenotype, chronicity, systemic spread, and treatment response. Arrows indicate activation; bar-headed lines indicate inhibitory mechanisms. Symbols beside a cell or cytokine indicate its current targetability by available drugs († targetable by JAK inhibitors; ‡ targetable by mTOR inhibitors; * targetable by HCQ; ** targetable by Methotrexate). Colors and patterning of lines and arrows are for visual differentiation purposes only; no correlation should be drawn between cells of similar color. Lines are solid or dashed for visual differentiation purposes only and do not indicate any inherent differences in pathogenesis. BAFF, B-cell activating factor; CD4, cluster of differentiation 4; CD8, cluster of differentiation 8; IFN-γ, interferon gamma; IL-4, interleukin 4; IL-6, interleukin 6; IL-13, interleukin 13; IL-17A, interleukin 17A; JAK, Janus kinase; JAK/STAT, Janus kinase/signal transducer and activator of transcription signaling pathway; mTOR, mechanistic target of rapamycin; mTORC1, mechanistic target of rapamycin complex 1; mTORC2, mechanistic target of rapamycin complex 2; NF-κB, nuclear factor kappa B; PD-1, programmed cell death protein 1; Rac1, Ras-related C3 botulinum toxin substrate 1; RhoA, Ras homolog family member A; SAA, serum amyloid A; STAT1, signal transducer and activator of transcription 1; STAT3, signal transducer and activator of transcription 3; TARC, thymus and activation-regulated chemokine (CCL17); Tfh, T follicular helper cell; TGF-β, transforming growth factor beta; Th1, T helper type 1 cell; Th2, T helper type 2 cell; Th17, T helper type 17 cell; Th17.1, interferon-γ–producing Th17 subset; TLRs, Toll-like receptors; Treg, regulatory T cell; TSC2, tuberous sclerosis complex 2. Created in BioRender. Grier, J (2026) https://BioRender.com/lwly9dw, licensed under CC BY 4.0.

Cutaneous sarcoidosis arises from dysregulated interactions between the adaptive and innate immune systems resulting in formation of epithelioid cell granulomas (**Figure 3**) (26). These non-caseating granulomas serve as an immune-mediated cellular barrier to isolate perceived foreign antigens and are composed of epithelioid histiocytes and multinucleated-giant cells encircled by lymphocytes, predominantly CD4^+^ T helper cells with occasional CD8^+^ T cells and B cells (27). Granuloma architecture is influenced by the predominant macrophage phenotype involved in the formation (26). Both M1 and M2 macrophages contribute to granuloma development through distinct, incompletely defined mechanisms (28). Th1-driven M1 macrophages release pro-inflammatory cytokines whereas Th2-driven M2 macrophages support tissue repair and may be involved in fibrosis (29, 30). While the M1/Th1 and M2/Th2 pathways are helpful for conceptualizing immune responses, it is important to note these are oversimplifications of a highly dynamic process. As such, granulomas often contain a heterogeneous mixture of cell phenotypes, rather than a single polarization pattern.

Given the immunopathology of the disease, the contemporary treatment of cutaneous, pulmonary, and systemic sarcoidosis relies on pharmacotherapy that reduces granulomatous inflammation. Prednisone and corticotropin gel are the only FDA-approved therapies for sarcoidosis, including cutaneous sarcoidosis (2). Prednisone, a glucocorticoid, works by inhibiting proinflammatory cytokine production and suppressing migration of polymorphonuclear leukocytes (31). While glucocorticoids are widely accepted as the first-choice pharmacotherapy for sarcoidosis treatment, refractory cases require the use of different agents. For glucocorticoid-refractory cutaneous sarcoidosis, or for patients with intolerance or comorbid diagnoses, cutaneous granulomas may respond to other immunosuppressants, particularly those that target dysregulated lymphocyte signaling. Through an increased understanding of the immunopathologic mechanisms of cutaneous sarcoidosis, novel therapeutic interventions for this disease can be discovered to provide positive impacts on patient care.

## 3 Cellular immunopathology

### 3.1 Th1 and Th17 cells

Emerging data from pulmonary sarcoidosis indicate that the classical Th1-centric model of non-caseating granuloma formation incompletely reflects the immunopathogenesis of sarcoidosis. In pulmonary sarcoidosis, a distinct CD4^+^ T cell subset, Th17.1 cells, have been identified as a major source of IFN-γ with marked expansion of IFN-γ-only producing Th17.1 cells, suggesting prior misclassification as Th1 cells and challenging the conventional Th1 paradigm (32). In blood and bronchoalveolar lavage (BAL) fluid of sarcoidosis patients with pulmonary involvement, IL-17A^+^IFN-γ^+^ memory Th cells are enriched compared with healthy controls, providing support for cooperative roles of IL-17 and IFN-γ in granuloma induction and maintenance (33). This has not yet been investigated in cutaneous sarcoidosis, but we postulate that a similar mechanism may be present in cutaneous sarcoid disease as IL-17A, produced by Th17 cells, is highly expressed within cutaneous sarcoid granulomas (34). Given the established contribution of Th17 pathways to other inflammatory and autoimmune diseases, further delineation of Th17 and Th17.1 subsets in cutaneous sarcoidosis is warranted.

### 3.2 JAK/STAT signal transduction

The Janus kinase/signal transducers and activators of transcription (JAK/STAT) pathway is one signaling pathway implicated in the formation of non-caseating granulomas in cutaneous sarcoidosis. In cutaneous sarcoidosis patients relative to healthy controls, immunohistochemical staining demonstrated significantly increased STAT1 and STAT3 phosphorylation, indicating pathway activation in lesional tissue, and this was further confirmed in tissue from sarcoidosis patients with skin or lung involvement (35, 36). In skin lesions from a patient with cutaneous sarcoidosis, JAK-STAT signaling was found to be particularly active as the genes encoding IL-6, the primary STAT3-activating cytokine, and IFN-γ, which activates STAT1, were highly expressed compared to normal skin (35, 37). Both IL-6 and IFN-γ were also elevated within pulmonary or site-undefined sarcoid granulomas with a predominance of Th1 and Th17 CD4^+^ T cells, suggesting a direct connection between JAK/STAT signaling and T cell dysregulation in sarcoidosis (36, 38, 39). These molecular signatures provide a rational basis for targeting JAK/STAT signaling to disrupt non-caseating granuloma formation in cutaneous sarcoidosis (35).

JAK inhibitors work to block production of numerous cytokines which may contribute to cutaneous sarcoidosis signaling pathways (2). A case report of glucocorticoid-refractory cutaneous sarcoidosis treated with tofacitinib, a JAK-inhibitor, resulted in clinical and histological improvements accompanied by decreases in IFN-γ and IL-6, thereby functionally linking JAK/STAT dysregulation to cutaneous sarcoidosis disease pathophysiology (35). In a subsequent case series, tofacitinib achieved complete or near-complete remission in three patients with cutaneous sarcoidosis, with improvement evident by two months and remission within six to ten months of therapy (40). Similar positive clinical outcomes were seen in two additional case reports of cutaneous sarcoidosis treatment with tofacitinib (41, 42). In a small clinical trial, treatment of cutaneous sarcoidosis with tofacitinib prompted clinical improvements which correlated with a reduction in the RNA expression of both IFN-γ and IL-6, as well as IL-2, IL-12, and IL-15, the expression of which are associated with Th1 responses (43). In other case reports, JAK/STAT inhibitors were also effective in the treatment of granuloma annulare and granulomatous rosacea, distinct cutaneous granulomatous diseases that have immunopathogenic mechanisms in common with cutaneous sarcoidosis (44–46). Collectively, these data suggest that aberrant JAK/STAT signaling is a major contributor to the pathogenesis of cutaneous sarcoidosis, with potential links to Th1/Th17 dysregulation, and presents a promising therapeutic target.

### 3.3 Th2 cells

Th2 cells, a subset of CD4^+^T helper cells, also participate in immune signaling pathways which may promote cutaneous sarcoidosis manifestations (29) and may even be associated with granuloma formation, as seen in some parasite infections or pulmonary sarcoidosis models (47, 48). Thymus and Activation-Regulated Chemokine (TARC/CCL17) is a Th2-associated chemokine and surrogate biomarker of Th2 activity (49). Nguyen et al. identified TARC expression in 100% of skin biopsies from sarcoidosis patients, with more intense TARC expression in mature cutaneous non-caseating granulomas relative to early-stage granulomas. Furthermore, TARC serum levels were elevated in 78% of sarcoidosis patients, with correlations to pulmonary disease severity (50). In addition to a strong association with Th2 cells, blockade of TARC in *ex vivo* cell cultures was also associated with a modest decrease in in IFN-γ and IL-17 production, suggesting that TARC may interact with Th1 and Th17 cells as well (51). This is further supported by confirmation of expression of CCR4, the TARC receptor, on Th17 cells (52). Thus, detection of TARC in serum and skin biopsies of sarcoidosis patients may serve as an effective biomarker for granulomatous inflammation mediated by either Th2 or Th17 cells. While TARC expression has not yet been investigated in isolated cutaneous sarcoidosis, its ubiquitous presence in cutaneous manifestations of systemic sarcoidosis implicates the Th2 pathways in cutaneous sarcoidosis immunopathology, particularly in latter disease stages. The Nguyen et al. study also found that a higher Th2:Th1 ratio was associated with greater disease severity and progression of cutaneous granulomas in systemic sarcoidosis as well as pulmonary sarcoidosis, supporting a role for Th2-skewed responses in non-caseating granuloma evolution at multiple sites, although the specific mechanisms at play have yet to be revealed (50).

### 3.4 Regulatory T cells

Regulatory T cells (Tregs), a CD4^+^ T cell subset essential for immune tolerance and suppression of excessive inflammation, are also dysregulated in sarcoidosis (53). Skin lesions from 16 patients with cutaneous sarcoidosis showed increased Tregs in the sarcoid granulomas, with Tregs found to predominantly localize to the periphery of cutaneous granulomas in another study of 9 patients with systemic sarcoidosis (54, 55). While there are conflicting reports regarding the total quantity of Tregs in peripheral blood in pulmonary and systemic sarcoidosis patients, reduced Treg suppressive function is thought to contribute to persistent inflammation and chronic disease (56–59). Limited capacity for Treg-mediated suppression, identified in sarcoidosis patients with pulmonary and/or renal involvement, may allow for initial granuloma formation and sustainment of immune mechanisms associated with granuloma maintenance (60, 61). Tregs from patients diagnosed with sarcoidosis specifically showed a reduced ability to suppress production of IFN-γ and the inflammatory cytokine TNF-α from other CD4^+^ cells (57). Tregs within pulmonary granulomas were even found to produce IL-4, a Th2 associated cytokine, which could contribute to chronic inflammatory processes (61). The ratio and location of Tregs relative to other CD4^+^ cells may also be an important indicator of disease state and clinical presentation. Among patients diagnosed with sarcoidosis, increased percentages of Tregs were identified in peripheral blood and those increases were positively associated with disease activity and severity (57–59). However, in bronchoalveolar lavage fluid, a lower frequency of Tregs paired with an increased proportion of Th17.1 cells was found in sarcoidosis patients relative to patients with idiopathic pulmonary fibrosis, suggesting a unique sarcoid-specific immune microenvironment in pulmonary sarcoidosis (59). Interestingly, a polymorphism associated with enhancer activity related to the Treg transcription factor FOXP3, was found to have no impact on systemic sarcoidosis susceptibility but appeared to provide an element of protection against cutaneous granuloma formation in patients with sarcoid disease (62). Thus, the role of Tregs in the pathogenesis of sarcoidosis may be quantity and location-specific, with differing impacts of Tregs in cutaneous sarcoidosis compared to pulmonary or systemic sarcoidosis, or even across different disease sites within a single patient.

An additional aspect of Treg biology that has not yet been studied in cutaneous sarcoidosis is the existence and significance of Treg subsets. A recent study of 28 patients diagnosed with sarcoidosis, with >50% of patients having documented lung involvement, found an increase in activated Tregs in the peripheral blood and those Tregs could then be further functionally categorized into suppressive effector Tregs or non-suppressive Tregs. Patients with a greater proportion of effector Tregs were more likely to experience spontaneous remission of sarcoid disease while patients who had experienced a relapse of symptoms were found to have a higher proportion of non-suppressive Tregs (63). Thus, future studies into Treg abundance, location, and subset functions in cutaneous sarcoidosis may provide significant insight into disease mechanisms and patient outcomes.

### 3.5 The PD-1 pathway

Programmed cell death protein 1 (PD-1) inhibits T-cell activation and promotes self-tolerance, in part, by inhibiting Treg apoptosis (64). Data directly linking PD-1 function and cutaneous sarcoidosis is limited, however insight from studies of non-cutaneous sarcoidosis may provide clues to the mechanisms driving cutaneous sarcoidosis immunopathology. In systemic sarcoidosis, an increased proportion of PD-1^+^ CD4^+^ T cells is associated with reduced T-cell proliferative capacity indicating dysregulated PD-1 signaling, relative to healthy controls (65). PD-1 signaling also modulates collagen production. The ability of pulmonary sarcoidosis CD4^+^ T cells to induce collagen-1 production by human fibroblasts *in vitro* is abrogated by PD-1 blockade (66). Upregulation of PD-1 on human Th17 cells promotes a profibrotic environment through IL-17A and TGF-β production by PD-1^+^CD4^+^ T cells (66). When taken in consideration with the elevated Treg counts and IL-17A expression observed in cutaneous sarcoidosis, these findings implicate PD-1 activity in cutaneous collagen deposition and sarcoidosis pathogenesis.

Despite the theoretical potential for PD-1 blockade to impede cellular mechanisms implicated in sarcoidosis, multiple case reports and series indicate that PD-1 inhibitors can precipitate *de novo* or recurrent cutaneous sarcoidosis as an immune related adverse event (67). Pembrolizumab and other PD-1 inhibitors have been associated with sarcoidosis flares involving the skin, lungs and eyes, typically arising within months of therapy initiation (67, 68). The pathogenesis of checkpoint inhibitor–associated sarcoidosis or sarcoid‑like reactions remains incompletely defined but likely reflects perturbation of complex Th1/Th17 responses and Treg pathways under immune activation conditions rather than simple loss of PD‑1–mediated inhibition (69–71). Paradoxically, both PD‑1 upregulation with reduced T‑cell proliferation in idiopathic sarcoidosis and exaggerated autoimmunity with PD‑1 blockade have been implicated in granuloma formation, underscoring the bidirectional and context‑dependent role of PD‑1 signaling in sarcoidosis pathobiology (65, 70, 71). These observations highlight the immunologic complexity of PD‑1 modulation in cutaneous sarcoidosis and suggest that therapeutic manipulation of this pathway may produce unanticipated clinical consequences.

## 4 Innate immune and intrinsic contributions to granuloma formation

### 4.1 Dysregulation of autophagy and mTOR signaling

Autophagy is an evolutionarily conserved process to maintain cellular homeostasis in which lysosomes break down and recycle dysfunctional biomolecules, organelles, and intracellular pathogens (72). While there are no direct studies linking autophagy to cutaneous sarcoidosis immunopathogenesis, mechanistic studies and a cutaneous sarcoidosis clinical trial provide strong evidence of an association between autophagy dysregulation and non-caseating granuloma formation. Autophagy is regulated by several signaling pathways, with mammalian target of rapamycin (mTOR) serving as the primary control mechanism (72–75). mTOR is a serine-threonine kinase that nucleates two protein complexes, mTORC1 and mTORC2, that promote cell growth and inhibit autophagy in response to plentiful ATP, amino acids, insulin and growth factors (76). During cellular stress or nutrient depletion, mTOR is inhibited and autophagy is induced (72, 77). However, in disease states where mTOR is highly expressed or unregulated, the resulting inhibition of autophagy may lead to a buildup of cellular waste, promoting non-caseating granuloma formation (72).

The mTOR pathway is also activated during T lymphocyte activation (78). Deletion of mTORC1 directly inhibits both Th1 and Th17 cells but preserves Th2 cell differentiation, while deletion of mTORC2 results in loss of Th2 cells with preservation of Th1 and Th17 differentiation (79). Taken together these data suggests that both mTORC1 and mTORC2 specifically contribute to the development of CD4^+^ T cell subsets associated with non-caseating granuloma formation and may promote sarcoidosis progression in affected patients (80).

Autophagy can also be triggered by Rac1, a constitutively expressed member of the Rho GTPase family, which may interact with mTOR to alter expression of genes associated with cell growth and change (80–82). The functions of Rac1 can be negatively regulated by RhoA, another Rho GTPase family member (83). Thus, dysregulation of mTOR or Rac1-associated pathways, due to mutation or negative regulation, may contribute to non-caseating granuloma formation via reduced pathogen and debris clearance as well as T cell and macrophage dysfunction (80, 84).

There is growing evidence that autophagy is a relevant consideration in the pathogenesis and treatment of conditions characterized by non-caseating granulomatous inflammation (26, 72). In mouse models, deletion of the mTOR inhibitor gene *Tuberous Sclerosis Complex Subunit 2* (*TSC2*), led to spontaneous formation of non-caseating granulomas with histopathologic similarity to systemic sarcoidosis granulomas (72, 80). Moreover, granulomatous phenotypes can be reversed with mTOR inhibitors, highlighting the association between mTOR activation and non-caseating granuloma formation (80, 85).

These studies of mTOR dysregulation highlight the potential utility of mTOR inhibitors in cutaneous sarcoidosis treatment protocols. In a single-center randomized clinical trial, the efficacy of the mTOR inhibitor sirolimus was evaluated as a treatment for patients with persistent and glucocorticoid-refractory cutaneous sarcoidosis. Sarcoidosis skin symptoms were measured using the Cutaneous Sarcoidosis Activity and Morphology Index (CSAMI), a qualitative tool designed to assess disease activity (85–87). Following systemic administration of sirolimus, seven of ten patients showed a CSAMI decrease of greater than five points and were categorized as responders (85). In the responders, sirolimus treatment was effective across various cutaneous sarcoidosis morphologies, including papules, nodules, plaques, and tattoo- and scar-associated sarcoidosis (85). Histologic analysis of skin biopsies revealed granuloma resolution or a substantial decrease in dermal granuloma size across all responders (85). Improvements in cutaneous sarcoidosis symptoms persisted through long-term follow up at two years, with no relapses recorded (85). Although small, this clinical trial provides valuable insights into the potential efficacy of mTOR inhibitors against granulomatous inflammation in cutaneous sarcoidosis (85).

Another signaling mechanism with autophagy-regulation capabilities is the aforementioned JAK/STAT pathway. The observed increase in JAK/STAT signaling in sarcoid-affected tissue is sufficient to inhibit autophagy and drive non-caseating granuloma formation (35, 88, 89). Thus JAK-STAT pathway inhibitors may prove to be effective treatments for cutaneous sarcoidosis due, in part, to an impact on autophagy signaling.

### 4.2 TLR signaling

Toll-like receptors (TLRs) are thought to be implicated in the inflammatory cascade of cutaneous sarcoidosis; however, studies investigating TLR blockade as a sarcoidosis treatment remain limited. TLR pattern recognition receptors initiate innate immune responses by detecting pathogen-associated and damage-associated molecular patterns. This recognition triggers intracellular signaling cascades that induce expression pro-inflammatory mediators such as nuclear factor kappa B (NF-κB), type I interferon (IFN), and inflammasome signaling (53, 90). In cutaneous sarcoidosis, immunohistochemical analysis demonstrated increased expression of TLRs 2, 3, 4, 5, 6, 7, and 8 in the dermis and epidermis of cutaneous sarcoidosis lesional skin, with TLR5 and TLR6 showing the most pronounced upregulation (91). Furthermore p65, an NF-κB signaling end product was present at high levels in cutaneous sarcoidosis granulomas (91). These findings implicate TLRs, especially TLR5 and TLR6, as potential therapeutic targets in cutaneous sarcoidosis. Multiple small-molecule and biologic TLR inhibitor drugs, including antagonists of TLR2, TLR4, TLR7, and TLR9 as well as downstream proteins in TLR signaling cascades, are currently under preclinical or clinical evaluation for inflammatory and autoimmune diseases (92) but have not yet been investigated specifically in the context of cutaneous sarcoidosis.

## 5 Humoral immunity in sarcoidosis

While sarcoidosis was previously thought to be primarily a cellular immunity-mediated disease, a growing body of evidence in the context of systemic sarcoidosis suggests that humoral pathway changes may be involved in cutaneous sarcoidosis pathogenesis. Associations of sarcoid disease with hypergammaglobulinemia, immune complex formation, and autoantibody production suggest a link between B cell dysregulation and symptomology (93, 94). In patients with severe chronic systemic sarcoidosis, investigation of B-cell subpopulation distribution detailed absolute B-cell lymphopenia, with significant reductions in CD19^+^ and CD27^+^ memory B-cells that was not due to localization or sequestration of CD20^+^ B cells in cutaneous granulomatous lesions (93–95). B-cell activating factor (BAFF) is a cytokine responsible for inhibiting B-cell apoptosis and promoting plasma cell differentiation. Cutaneous sarcoidosis granulomas have been found to produce BAFF and may contribute to increased serum BAFF levels in sarcoid disease states (94). Notably, in patients with systemic sarcoidosis, serum BAFF levels are significantly increased, suggesting that BAFF expression may align with disease activity (93, 94, 96). In patients with elevated serum BAFF, there is also a higher likelihood of cutaneous and ocular granulomatous involvement (94).

While these studies support a role for B-cells in the pathogenesis of sarcoidosis, the utility of B-cell targeted therapies remains unclear. Rituximab, an anti-CD20 monoclonal antibody, has been used in the treatment of refractory systemic sarcoidosis with varied responses (97–99). There is also mixed efficacy in case reports of rituximab use in cutaneous disease, ranging from no response to complete resolution of skin lesions (100). In contrast, other reports have detailed cutaneous and extracutaneous sarcoidosis as an adverse event in the setting of rituximab therapy (101–103) The mechanism of this drug-induced sarcoid reaction is not known but may be a consequence of B cell depletion leading to a loss of regulatory interactions between B and T cells (104). The BAFF inhibiting monoclonal antibody, belimumab, is currently approved for the management of systemic lupus erythematosus (SLE) and may represent a potential therapeutic option for patients with recalcitrant sarcoidosis, but it has not yet been studied in cutaneous sarcoidosis (105, 106). With dysregulated antibody production in sarcoid disease and elevation of BAFF in cutaneous non-caseating granulomas, it seems likely that B cells contribute to cutaneous sarcoidosis immunopathology. However, further studies are needed to fully evaluate the utility of B-cell targeted therapies in the management of cutaneous disease.

## 6 Additional contributors to cutaneous sarcoidosis susceptibility

### 6.1 Infectious agents

Various studies have described the role of infectious agents in the pathogenesis of cutaneous sarcoidosis, most notably *Mycobacterium* spp. Analysis of 20 cases of cutaneous sarcoidosis revealed *Mycobacterium* spp. DNA in 80% of patients, with *M. tuberculosis* complex, *M. avium-intracellulare*, *M. kansaii* or non-tuberculous *Mycobacterium* (NTM) detected in cutaneous lesions (107). *Mycobacterium* heat shock proteins (HSPs) were also detected in cutaneous sarcoidosis lesions, consistent with the presence of *M. tuberculosis*, *M chelonae*, and *M. gordonae* DNA (108). A recent study identified both *M. tuberculosis* and NTM in 6 of 9 patients with cutaneous sarcoidosis (109). Strengthening the argument for an infectious etiology of sarcoidosis, various case reports and clinical trials have reported resolution of cutaneous lesions following antimicrobial therapy (110–112). A randomized placebo-controlled trial of 30 chronic cutaneous sarcoidosis patients treated with oral antimycobacterial therapy found decreased lesion diameter, reduced non-caseating granuloma burden, and diminished lesion severity (110). These findings suggest an infectious etiology in the pathogenesis of sarcoidosis and highlight the possible role of antimycobacterial therapies, alone or in combination with immunomodulators, in the treatment of cutaneous sarcoidosis specifically.

### 6.2 DNA polymorphisms

Genome-wide-association studies (GWAS) of patients with sarcoidosis also indicate that genetic predisposition may contribute to cutaneous sarcoid granuloma development. GWAS and transcriptome wide association studies (TWAS) identified 28 loci associated with any sarcoidosis diagnosis regardless of site (113). Loci near to immune-associated genes including *major histocompatibility complex* (*MHC*), *interleukin-23* (*IL23*), *coiled-coil domain containing protein 88B* (*CCDC88B*), *annexin A11* (*ANXA11*), and *nucleotide-binding oligomerization domain-containing protein 2* (*NOD2*) were among those linked to sarcoidosis (113). The largest effect size was found with the *interleukin-23 receptor* (*IL23R)* locus which has previously been linked with sarcoidosis in African American and East Asian populations; thus, targeting of IL-23 signaling could be useful as a therapeutic approach in the future (113, 114). Gene expression of CCDC88 and Tyrosine Kinase 2 (TYK2) was also associated with sarcoidosis disease susceptibility (113). Several human leukocyte antigen (HLA) polymorphisms of HLA-DRB1 and HLA-DQB1 were found to be related to various clinical forms of sarcoidosis (115). A strong association was identified between the HLA-DRB1*0301 allele and Lofgren’s syndrome, an acute sarcoid disorder, in Dutch patients (115). Whereas, an association between HLA-DRB1*15 and HLA-DQB1*0602 alleles and sarcoidosis with cutaneous involvement was identified in patients from Japan (115). As the relationships between genetic variability and specific susceptibility to cutaneous manifestations of sarcoidosis are revealed, targeted genetic therapies may represent a future area of discovery and innovation for treatment of affected patients.

## 7 Therapeutic alternatives for the treatment of cutaneous sarcoidosis and safety considerations

Available therapeutic options for the treatment of cutaneous sarcoidosis vary significantly in terms of specific targets and the strength of the supporting evidence on both mechanistic and clinical levels (**Table 3**). In many cases, the direct evidence for treatment of cutaneous sarcoidosis is lacking or limited to case studies or small trials. In the United States, there are currently no immunosuppressive biologics approved by the Food and Drug Administration for the treatment of sarcoidosis, which limits their use to “off label” regimens that may not be covered by a patient’s insurance, creating an additional hurdle to treatment (116). Yet for cutaneous sarcoidosis patients with prednisone-associated contraindications or glucocorticoid-refractory cutaneous sarcoidosis, these therapeutic alternatives may provide a means to reduce or resolve their disease symptoms. The use of non-steroidal therapeutics and targeted immunosuppressive medications to treat moderate to severe sarcoidosis is an accepted component of treatment algorithms across a number of treatment guidelines (117, 118). As with any pharmacotherapy, it is also important to consider safety and potential adverse events when selecting a patient treatment regimen for cutaneous sarcoidosis.

**Table 3:** Existing and novel therapeutics for lymphocyte signal dysfunction in cutaneous sarcoidosis.

Drug	Target	Evidence for use in cutaneous sarcoidosis
Prednisone	glucocorticoid receptor	first-line therapy for extra-pulmonary sarcoidosis extrapolated to cutaneous (117)
Infliximab/adalimumab	TNF-α	randomized clinical trial of 10 patients (121) or 12 patients (120); Retrospective review of 9 or 46 patients (119, 120, 122)
Methotrexate	dihydrofolate reductase	studies of 3–23 patients with >80% response rate of cutaneous disease (127–130)
Hydroxychloroquine	lysosomes, TLR3, TLR7, TLR9	clinical trial of 17 patients (137); Retrospective review of 13 sarcoidosis patients with cutaneous involvement (154)
Tofacitinib	JAK1/JAK3	case reports (35, 41, 42); Case series of 3 patients (40); Clinical trial of 10 patients (43)
Sirolimus	mTOR	clinical trial of 16 patients (85)
Rituximab	B cells	case reports of systemic sarcoidosis or neurosarcoidosis with cutaneous involvement (155, 156), clinical trial of refractory pulmonary sarcoidosis in 10 patients (98), retrospective review of 4 patients with refractory ocular sarcoidosis (157) and 19 patients with neurosarcoidosis (99) extrapolated to cutaneous sarcoidosis

TNF-α, Tumor necrosis factor-α; TLR, Toll-like Receptor; JAK, Janus Kinase; mTOR, mammalian Target of Rapamycin.

TNF-α inhibitors, most commonly infliximab and adalimumab, are often used as third-line therapy for refractory, progressive, or organ-threatening sarcoidosis, particularly when corticosteroids and conventional immunosuppressants are inadequate. By blocking TNF-α, a key cytokine in macrophage activation and granuloma maintenance, these agents can reduce granulomatous inflammation. Infliximab has shown positive results in multiple small studies of patients with refractory cutaneous sarcoidosis (119, 120). While clinical and retrospective studies show meaningful improvements in roughly two-thirds to three-quarters of patients with severe or cutaneous sarcoidosis, often allowing steroid dose reduction, responses may take months and relapse can occur after discontinuation (121, 122). The use of TNF-α inhibitors is limited due to a high risk of infection, among other adverse effects, so these drugs are reserved for serious or treatment-resistant disease. Paradoxically, a small percentage of patients receiving TNF-α inhibitors may develop a drug induced sarcoid reaction, hypothetically due to a compensatory increase of other inflammatory cytokines such as IFN-γ or Th1 skewing of T cell differentiation (123, 124).

Sarcoidosis patients who fail prednisone may move to anti-metabolites such as Methotrexate, Azathioprine, Leflunomide, or Mycophenolate Mofetil, with Methotrexate being the preferred choice (125). Methotrexate inhibits dihydrofolate reductase and prevents DNA synthesis, while also disrupting adenosine and guanine metabolism (126). This leads to adenosine accumulation that prevents T-cell activation and increases the sensitivity of T-cells to apoptotic signals, ultimately reducing inflammatory signaling (126). To reduce prednisone use and minimize the many adverse side effects patients experience with prednisone, Methotrexate is often used off label for cutaneous sarcoidosis, with a >80% response rate in cutaneous disease across multiple studies (2, 127–130). Although the overall sample population of these studies is limited, the mechanism of action and high response rates suggest that Methotrexate could be an efficacious treatment for cutaneous sarcoidosis-associated inflammation.

Hydroxychloroquine (HCQ), brand name Plaquenil, was originally used as an antimalarial drug, but was found to improve rashes and inflammatory arthritis in soldiers taking HCQ for malaria prophylaxis (131). Today, HCQ is used in the treatment of many autoimmune and autoinflammatory diseases including cutaneous sarcoidosis, due to a variety of anti-inflammatory and immunomodulatory effects (132). HCQ impairs antigen processing and presentation by MHC-II complexes contributing to a decrease in cell mediated immune responses (133). HCQ also blocks signaling of TLR3 and TLR7, leading to decreased pro-inflammatory cytokines, including IFN-γ (131). Additionally, HCQ reduces Th17 cell counts with decreased IL-6, IL-17, and IL-22 as a result (134, 135). HCQ treatment for dermatologic disorders appears promising despite small sample sizes (136). In a clinical trial, 12/17 patients with cutaneous sarcoidosis had regression with HCQ and were able to stop other medications while a further 3/17 patients had a partial response (137). Thus, HCQ offers an alternative to corticosteroids in the treatment of cutaneous sarcoidosis.

Currently, JAK inhibitors are in preliminary investigation for the treatment of cutaneous sarcoidosis. Case report and clinical trial data in a small number of patients with cutaneous sarcoidosis demonstrated that treatment with the JAK1/JAK3 inhibitor tofacitinib can dampen inflammatory signaling and reduce disease symptoms (35, 43). Unlike JAK1 and JAK2, which are ubiquitous, JAK3 is primarily expressed in hematopoietic cells, which may provide some insight into the efficacy of tofacitinib in reducing lymphocyte-mediated granulomatous inflammation (138). JAK inhibitors may also have a therapeutic advantage over existing treatments as tofacitinib was able to induce clinical and histologic remission in refractory cutaneous sarcoidosis cases (35, 40–42). Other JAK-inhibitors such as ruxolitinib and baricitinib, which target JAK1/JAK2, have been identified as treatment options for refractory cutaneous or pulmonary sarcoidosis (40); however, data on these treatments remains limited to case reports, small retrospective series, or early trials (139, 140). There are important safety considerations for use of JAK inhibitors including an increased risk of serious infections, cardiovascular events, venous thromboembolism, dyslipidemia, gastrointestinal perforation, malignancy, and death (141, 142). JAK inhibitor use may be associated with reactivation of herpes zoster, tuberculosis, opportunistic infections, and upper respiratory infections (142). Monitoring of hepatic and renal function, as well as routine screenings for tuberculosis, hepatitis, cancer, and diverticular disease are imperative for patients taking JAK inhibitors. Given the elevated levels of JAK-STAT in sarcoid cells relative to controls, there is potential for JAK-STAT pathway inhibitors to slow the progression of cutaneous sarcoidosis through a combination of T cell inhibition and release of JAK-mediated autophagy inhibition (35, 88, 89).

Additional immunomodulatory therapies such as mTOR inhibitors and TLR inhibitors show promise as cutaneous sarcoidosis therapeutics due to targeting of intracellular and pro-inflammatory mechanisms that contribute to cutaneous sarcoidosis disease; however, these treatments are largely still theoretical (26, 92). While inhibitors of TLR signaling and downstream targets have been evaluated for use in immunopathogenic disorders such as Systemic Lupus Erythematosus, there are no cutaneous sarcoidosis studies to date (92). A small study of the mTOR inhibitor sirolimus showed improvement in seven out of ten participants with cutaneous sarcoidosis, which is promising despite the small sample size (85). Important safety considerations for use of mTOR inhibitors include significant risks for stomatitis, metabolic derangements, myelosuppression, infections, and impaired wound healing (143). Other important risks include rash, fever, fatigue, diarrhea, elevated liver enzymes, acute tubular necrosis, proteinuria, and hypertension (144). Non-infectious pneumonitis, although rare with mTOR inhibitor use, can be fatal (145). Physicians overseeing the care of patients taking mTOR inhibitors should monitor for new-onset respiratory symptoms and evaluate to exclude possible bacterial pneumonia and opportunistic infections (146). Close monitoring of hematologic markers, lipids, hepatorenal function, and glucose are also required.

B-cell blockers such as Rituximab are also being investigated for refractory sarcoidosis (147). In a few case reports, success with Rituximab was noted for patients with refractory sarcoidosis, but a small prospective open-label trial of patients with refractory pulmonary sarcoidosis found no benefit (147). Safety considerations for B-cell blocker treatments include a risk of fatal infusion-related reactions, severe mucocutaneous reactions, including Stevens-Johnson syndrome and toxic epidermal necrolysis, hepatitis B virus reactivation, and progressive multifocal leukoencephalopathy (148). Other serious risks include tumor lysis syndrome, infection, adverse cardiovascular reactions, renal toxicity, bowel obstruction and perforation, and fetal toxicity. Potentially fatal pulmonary toxicity can also occur in patients taking Rituximab (145). Patients being treated with rituximab should be monitored closely for new-onset infection, arrhythmias, renal failure, abdominal pain, pregnancy, and cytopenias (148).

Therapeutic options for cutaneous sarcoidosis treatment continue to expand to include new immunosuppressant therapies and monoclonal antibodies. Advances in the mechanistic understanding of cutaneous sarcoidosis continue to reveal new pathways and targets for disease treatment. As the body of scientific knowledge regarding the immune mechanisms underlying cutaneous sarcoidosis inflammation and granuloma formation continues to grow, new insights can be applied in clinical settings to guide the selection of therapeutics. Given the concerted effects of multiple T cell types, autophagy and mTOR signaling, TLR signaling, and humoral immunity in the development of the sarcoid disease, emerging therapeutics that inhibit multiple arms of the immune system may prove most effective in the treatment of patients with cutaneous sarcoidosis. Evidence for this approach may already exist in the efficacy of corticosteroids as a first line of treatment for sarcoidosis. Future research may reveal similar efficacy with JAK and mTOR inhibition as these pathways contribute to both innate and adaptive immune mechanisms at play in cutaneous sarcoidosis. While large scale clinical trials are ultimately needed to fully determine safety and efficacy, further understanding of the signaling mechanisms driving pathogenesis of cutaneous sarcoidosis will continue to open up new avenues for novel therapeutics for this dysregulated immune disease.

## 8 Conclusions and future perspectives

The T-cell-centric model of immune signaling for non-specific non-caseating granuloma formation does not fully encompass the pathogenesis of cutaneous sarcoidosis. Under-discussed cellular mechanisms which contribute to the pathophysiology of cutaneous sarcoidosis include increased expression of PD-1, TLR signaling, B cell lymphopenia, presence of mycobacterium and DNA polymorphisms. The JAK/STAT pathway, mTOR regulation, and dysfunction of autophagy have also been linked to the formation of granulomas in the pathophysiology of sarcoidosis.

Elucidation of the cell signaling mechanisms at play in the pathogenesis of cutaneous sarcoidosis is crucial as it will allow for the development of novel therapeutics to combat the disease (2). Although sarcoid-specific skin lesions share the same core immunopathology of non-caseating granulomas driven by Th1/Th17-mediated macrophage activation, the relative intensity, chronicity, and downstream immune skewing differ across morphologies and influence therapeutic management (149). The superficial, inflammation-dominant immune activity and limited fibrosis of small papules and lesions often respond to topical or intralesional corticosteroids and antimalarials, with a greater likelihood of remission (14, 150). In contrast, more persistent cutaneous granulomatous inflammation with evolving Th2/M2 macrophage and fibrotic signaling is often less responsive to local therapy and more likely to require systemic immunomodulators (147). The cutaneous sarcoidosis presentation of Lupus pernio lies at the chronic, high-burden end of the spectrum, characterized by sustained Th1/Th17 activation, macrophage-driven remodeling, and fibrosis. It is frequently refractory to topical therapy, often necessitating early and aggressive systemic treatment, or targeted biologic agents (151, 152). Thus, lesion morphology reflects where disease lies along the inflammation–chronicity–fibrosis axis and helps guide the intensity of therapy rather than indicating fundamentally different pathogenic pathways.

These morphological considerations paired with cutaneous sarcoidosis signaling insights can guide practicing clinicians in providing treatment recommendations for affected individuals. Prednisone and corticotropin gel are currently the only FDA approved therapies for sarcoidosis, including cutaneous sarcoidosis; however, there are several therapeutics under investigation based on sarcoidosis cell-signaling research. If prednisone cannot be used or fails to resolve cutaneous sarcoidosis lesions, methotrexate and hydroxychloroquine may be prescribed as the next line of treatment and have proven successful in small-scale studies of cutaneous sarcoidosis. Studies investigating JAK inhibitors, mTOR inhibitors, and rituximab have led to significant advances in the development of novel treatment regimens for cutaneous and systemic sarcoidosis, but further research must be completed before these therapeutics can be broadly applied for patient use. As the understanding of lymphocyte signaling pathways at the heart of cutaneous sarcoidosis disease mechanisms continues to grow, new avenues for effective treatments will open and ultimately improve patient outcomes for those affected.

### 8.1 Clinical implications

- Specific skin manifestations should be considered when choosing cutaneous sarcoidosis therapy regimen in order to match immunotherapeutic mechanisms to the immunopathological state of the lesion.
- In small studies, JAK/STAT inhibitors have proven effective for cutaneous sarcoidosis treatment likely due to a dual mechanism impacting T cell activation and promotion of autophagy.
- Large scale studies of mTOR inhibitors and methotrexate should build upon previous small studies with successful patient responses.
- TLR inhibitors may prove effective as cutaneous sarcoidosis treatment approaches in the future, via inhibition of early Th1 and Th17-associated granuloma formation, but extensive testing is still needed.
- The clinical utility of PD-1 inhibitors and B cell targeted therapies remains unclear due to mixed results in small clinical studies paired with high risks of adverse events.

### 8.2 Research priorities

- Identification of biomarkers present in cutaneous sarcoidosis lesions that are predictive of systemic inflammatory or sarcoid states.
- Investigation of the contributions of Th17.1 cells to cutaneous sarcoidosis disease manifestations.
- Discernment of the contributions of T regs and PD-1 signaling in cutaneous sarcoidosis disease presentation compared to pulmonary or systemic sarcoidosis.
- Investigation of the functions of mTOR and Rac1 in cutaneous sarcoidosis lesions in relation to Th cell subset differentiation and autophagy.
- Evaluation of antimicrobial therapies as part of a combined drug regimen that includes immunomodulatory drugs for treatment of cutaneous sarcoidosis.
